# Trends of main indicators of leprosy in Brazilian municipalities with high risk of leprosy transmission, 2001–2012

**DOI:** 10.1186/s12879-016-1798-2

**Published:** 2016-09-05

**Authors:** Lucia R. S. Freitas, Elisabeth C. Duarte, Leila P. Garcia

**Affiliations:** 1 Programa de Pós-Graduação em Medicina Tropical, Núcleo de Medicina Tropical, Faculdade de Medicina, Universidade de Brasília, Brasília, Brazil; 2 Núcleo de Medicina Tropical, Faculdade de Medicina, Universidade de Brasília, Brasília, Brazil; 3 Instituto de Pesquisa Econômica Aplicada, Brasília, Brazil

**Keywords:** Leprosy, Epidemiology, Ecological studies, Trends, Surveillance

## Abstract

**Background:**

Leprosy incidence has reduced in recent years in Brazil, although the disease still persists as a public health problem in some regions. To investigate the trends of selected leprosy indicators in Brazilian municipalities with high risk of transmission is essential to provide effective control of the disease, yet this area has not been investigated.

**Methods:**

This is﻿ an ecological time-series study with multiple groups using Notifiable Diseases Information System (*SINAN*) data. All 692 municipalities of the states of Mato Grosso, Tocantins, Rondônia, Pará and Maranhão were included. The incidence rates of leprosy were calculated, as well as incidence rates in children under 15 years per 100,000 inhabitants and rates of new cases presenting grade-2 disabilities per 100,000 inhabitants. Joinpoint Regression was used to analyse the time trends of the different indicators studied. The spatial distribution of temporal variations of the indicators in the period was presented.

**Results:**

Between 2001 and 2012, 176,929 leprosy cases were notified in the area studied, this being equivalent to 34.6 % of total cases in Brazil. In the aggregate of municipalities, there was a reduction in incidence rate of leprosy from 89.10 to 56.98 new cases per 100,000 inhabitants between 2001 and 2012, with a significant reduction between 2003 and 2012 (APC: − 6.2 %, 95 % CI: −7.2 % to −5.2 %). The incidence rate in <15 years also reduced significantly between 2003 and 2012 (APC: −5.6 %; 95 % CI: −7.2 % to −4.1 %). The rate of new cases with grade 2 disability remained stable between 2001 and 2012 (APC: −1.3 %; 95 % CI: −2.6 % to 0.1 %).

**Conclusion:**

Despite the reduction in the leprosy incidence rate, strategies for controlling this disease need to be enhanced to enable early case detection, especially in hyperendemic municipalities, in order to prevent disability.

## Background

Leprosy persists as a significant health problem in several parts of the world. According to the official reports of 121 countries, 213,899 new cases were notified worldwide in 2014, 125,785 (59 %) of which occurred in India, 31,064 (15 %) in Brazil and 17,025 (8 %) in Indonesia. These countries accounted for 81 % of total new cases notified globally [1].

Between 1 and 2 million people worldwide are currently estimated to have deformities and disabilities resulting from leprosy and it continues to be one of the main causes of neuropathy and disabilities among communicable diseases [2].

In Brazil, in 2013, were notified 31,044 new cases of leprosy and 2,439 new cases in children under 15 years. The new case detection rate was 15.44 cases per 100,000 inhabitants, the rate of new cases with grade 2 disability was 0.99 per 100.000 inhabitants, and the detection rate in children under 15 years was 5.03 cases per 100,000 inhabitants [3].

Following the introduction of multidrug therapy (MDT), the achievement of high Bacillus Calmette-Guérin (BCG) vaccination coverage in children and improvements in disease control, leprosy prevalence in Brazil decreased substantially from 180 cases per 100,000 inhabitants in 1988, to 26 cases per 100,000 inhabitants in 2008 [4]. Despite efforts to reduce the prevalence of the disease, these actions have apparently little effect on the reducing transmission and incidence [4–6]. Use of leprosy prevalence as an indicator has been criticized since it is influenced by factors such as treatment duration and case identification. Alternative indicators for monitoring the disease have been suggested, such as the rate of new cases with grade 2 disability [2, 7].

The objective of this study is to describe the trends of the main indicators of leprosy in Brazilian municipalities with high risk of transmission in the period between 2001 and 2012.

## Methods

This i﻿s an ecological time-series study with multiple groups (spatial trends) [8] of selected epidemiological indicators used to monitor leprosy between 2001 and 2012. The units of analysis used by this study were municipalities according to inclusion criteria.

Brazil is divided into five regions (North, Northeast, Midwest, Southeast and South), 26 states and a Federal District. It is South America’s largest country (8,515,767 km^2^). In 2010 it had 190.7 million inhabitants [9].

**Fig. 1:**
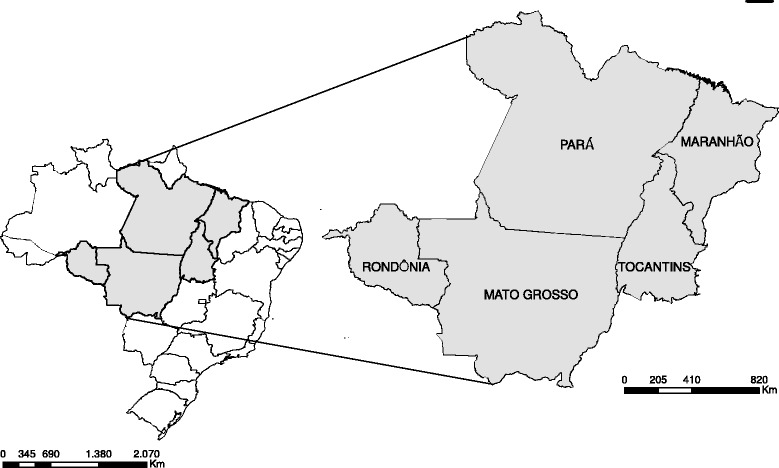
Study area: all municipalities of the states of Mato Grosso, Tocantins, Rondônia, Pará and Maranhão

All 692 municipalities of the states of Mato Grosso, Tocantins, Rondônia, Pará and Maranhão, located in the country’s North, Northeast and Center-West regions, were included in the analysis (Fig. 1). The study area covers 2,998,569 km^2^ and according to the 2010 demographic census it had a total population of 20.1 million inhabitants, accounting for 10.6 % of the Brazilian population. These municipalities are located in what is considered to be a high risk area for leprosy transmission, according to cluster analysis undertaken in 2009 by the Brazilian Ministry of Health [10]. Moreover, a study conducted in 2009–2011 found that 442 (48.4 %) of the total of 914 municipalities located in the Midwest and Northern regions had average incidence rates greater than 40.0 new cases per 100,000 inhabitants and were considered to be hyperendemic [11].

The data were obtained from the Notifiable Diseases Information System (*Sistema de Informação de Agravos de Notificação - SINAN*). *SINAN* is the main information system that records the compulsory notifications of several different diseases nationwide, including leprosy [12].

The following epidemiological indicators for leprosy were selected and calculated: incidence rate of leprosy per 100,000 inhabitants, incidence rate in children under 15 years per 100,000 children and rate of new case with grade 2 disabilities per 100,000 inhabitants. The criteria for grade 2 disability are: hands and feet: visible deformity or damage present; eyes: severe visual impairment (vision worse than 6/60), inability to count fingers at 6 meters), also includes lagophthalmos, iridocyclitis and corneal opacities [7]. In this study, we used the term incidence rate of leprosy as synonymous for the detection rate of leprosy, and implication of this is discussed latter in this manuscript.

These indicators were selected because they are used in monitoring leprosy by the control program in Brazil and for presenting the magnitude of occurrence of disease in the population. The size of the resident population used as the denominator was based on the 2010 Census and intercensal projections (2001–2012) produced by the Brazilian Institute of Geography and Statistics (IBGE) [13].

The parameter categories adopted by the Brazilian Ministry of Health [14] for interpreting each indicator were defined, namely: incidence rate of leprosy – low: <2.00 per 100,000 inhabitants; medium: 2.00 to 9.99 per 100,000 inhabitants; high: 10.00 to 19.99 per 100,000 inhabitants; very high: 20.00 to 39.99 per 100,000 inhabitants and hyperendemic: ≥40.00 per 100,000 inhabitants); incidence rate of leprosy in children under 15 years – low: <0.50 per 100,000 children; medium: 0.50 to 2.49 per 100,000 children; high: 2.50 to 4.99 per 100,000 children; very high: 5.00 to 9.99 per 100,000 children and hyperendemic: ≥10.00 per 100,000 children). With regard to the rate of new cases with grade 2 disability, the categories used were based on approximate quartiles of the number of cases notified in the periods 2001–2003 and 2010–2012 – without cases: 0; high: >0 to <4 cases and very high: ≥4 cases.

The rates were aggregated into four 3-year periods (2001–2003, 2004–2006, 2007–2009 and 2010–2012). Maps of the percentage change in the epidemiological indicators of leprosy were generated for the periods 2001–2003 and 2010–2012. Taking the periods 2001–2003 and 2010–2012, two groups of municipalities were created for each indicator in accordance with the parameters defined for interpreting them: municipalities with indicators showing increases (percentage change >20 %); and all other municipalities – those with indicators showing reduction, maintenance or increase not greater than 20 % (percentage change ≤20 %).

Joinpoint regression was used to calculate annual indicator variation between 2001 and 2012. This analysis method consists of segmented linear regression (joinpoint regression) to identify points where trends change and to estimate annual percentage change (APC) and average annual percentage change (AAPC) considering the entire period of the series. Successive models were adjusted whereby in each model there were assumed to be a different number of trend change “points”, ranging from zero (where the trend is represented by a single straight segment) up to no more than three, due to the quantity of observations. The model chosen was the one with the highest number of points maintaining statistical significance (*p* <0.05). Based on the estimated inclination for each straight segment (regression coefficient), annual change was calculated as a percentage and its statistical significance was estimated using the generalized linear model least squares method, assuming that rates follow Poisson distribution and that rate variation is not constant over time [15]. Confidence interval (95 % CI) limits were calculated for each straight segment (using estimated inclination). During trend analysis of the rates of new cases with grade 2 disability, the rate for the year 2007 was removed because in that year there was a change in the information system regarding the definition of grade 2 disability and this could have caused trend inconsistency. This decision regarding the method is justified more clearly in the results section (Table 1).

**Table 1:** Epidemiological indicators related to leprosy, according to the year of notification and states, 2001–2012

Indicator	State (number of municipalities)	2001	2002	2003	2004	2005	2006	2007	2008	2009	2010	2011	2012	Line graph
Incidence rates of leprosy per 100,000 inhabitants	Aggregated of municipalities (692)	89.10	94.08	98.16	95.29	91.25	81.63	72.05	73.17	66.52	61.95	60.87	56,98	
	Rondônia (52)	82.46	85.14	95.82	91.22	82.82	87.17	73.08	74.59	71.15	59.91	53.79	50,19	
	Pará (143)	80.97	90.65	92.46	89.47	77.45	69.38	60.94	63.46	55.54	49.29	50.53	49,50	
	Tocantins (139)	91.74	98.18	104.78	102.37	95.35	107.47	96.48	105.50	88.31	78.14	71.88	72,23	
	Maranhão (217)	78.07	82.30	86.13	89.08	91.90	75.37	66.81	68.76	62.87	62.33	59.26	53,84	
	Mato Grosso (141)	136.30	131.84	137.14	122.36	126.85	110.60	98.99	91.86	89.78	86.39	88.79	79,03	
	Other states of Brazil (4.873)	18.88	20.40	21.33	20.31	19.31	17.64	15.71	15.61	14.67	13.78	13.05	12,10	
Incidence rates of leprosy in clhildren <15 years per 100,000 inhabitants	Aggregated of municipalities (692)	24.32	26.40	27.80	26.30	25.47	21.41	20.22	21.43	19.74	18.35	18.48	15,79	
	Rondônia (52)	15.64	15.98	21.87	19.36	13.75	17.75	16.28	21.09	20.20	11.08	10.51	10,18	
	Pará (143)	25.68	27.57	28.62	28.22	24.05	20.32	19.99	21.54	19.07	17.20	17.41	15,21	
	Tocantins (139)	23.74	24.02	28.66	24.28	24.20	27.99	28.51	32.14	27.26	17.84	19.86	22,08	
	Maranhão (217)	21.96	25.57	25.77	25.07	28.42	22.62	18.89	19.05	19.22	20.70	19.36	16,22	
	Mato Grosso (141)	30.22	31.39	32.37	27.84	28.90	19.23	21.70	21.12	18.22	19.24	20.62	16,24	
	Other states of Brazil (4.873)	4.33	4.45	4.91	4.76	4.53	4.11	4.14	4.08	3.64	3.54	3.23	3,06	
Rates of new cases with grade-2 disabilities per 100,000 inhabitants	Aggregated of municipalities (692)	3.62	4.08	3.83	3.43	3.78	3.29	4.87	3.96	3.55	3.15	3.27	3,41	
	Rondônia (52)	2.77	4.33	6.94	4.46	6.00	4.80	4.34	5.49	4.12	3.14	2.47	3,46	
	Pará (143)	3.33	3.90	3.10	2.97	2.90	2.33	3.96	3.25	2.93	2.40	2.80	2,80	
	Tocantins (139)	3.04	4.81	3.82	4.87	4.29	3.75	6.77	3.98	3.87	3.54	4.35	3,88	
	Maranhão (217)	4.19	3.89	3.46	3.23	4.08	3.75	5.17	4.84	4.08	3.45	3.36	3,65	
	Mato Grosso (141)	3.83	4.45	4.79	3.74	3.89	3.64	5.88	3.08	3.53	4.22	4.16	4,21	
	Other states of Brazil (4.873)	1.12	1.16	1.19	1.22	1.16	1.09	1.32	1.21	1.06	1.01	0.94	0,86	

Analyses were performed with the aid of Joinpoint version 3.5.1 (Statistical Research and Applications Branch, National Cancer Institute, Rockville, MD, USA), SatScan 9.3 (Kulldorff 2014), R 3.0.2 (R Core Team 2013) and ArcGis 9.2 (Environmental Systems Research Institute, Redlands, CA, USA) (ESRI 2010).

The Ethics Committee of the Health Sciences Faculty of the University of Brasília approved the project under number Presentation of Certificate for Ethics Assessment (CAAE) 20249613.9.0000.0030.

## Results

Between 2001 and 2012, a total of 176,929 cases of leprosy were notified in the municipalities studied, accounting for 34.6 % of all new leprosy cases in Brazil in 10 % of the country’s population. In the aggregate of municipalities, there was a reduction in incidence rate of leprosy from 89.10 new cases per 100,000 inhabitants in 2001 to 56.98 new cases per 100,000 inhabitants in 2012 (Tables 1 and 2). This reduction was statistically significant in the period 2003–2012 (APC: −6.2 %, 95 % CI: −7.2 to −5.2 %; Table 2). In all five states studied, there was also statistically significant reduction in incidence rates of leprosy, especially the state of Tocantins from 2008 to 2012 (APC: −9.7 %, 95 % CI: −15.2 to −4.0 %), and the state of Mato Grosso from 2001 to 2012 (APC: −6.2 %, 95 % CI: −7.2 to −5.2 %) (Tables 1 and 2).

**Table 2:** Joinpoint regression analysis of epidemiological indicators related to leprosy, 2001–2012

Indicator	State	Annual percentage change (APC)			Average annual percentage change (AAPC)		
		Period	APC	95 % CI	Intire period	AAPC	95 % CI
Incidence rates of leprosy per 100,000 inhabitants	Aggregated of municipalities	2001–2003	5.8	−5.3 a 18.1	2001–2012	−4.2*	−5.9 a −2.4
		2003–2012	−6.2*	−7.2 a −5.2			
	Rondônia	2001–2004	4.7	−4.7 a 14.9	2001–2012	−4.1*	−6.5 a −1.7
		2004–2012	−7.2*	−9.3 a −5.1			
	Pará	2001–2003	5.2	−13.1 a 27.3	2001–2012	−5.3*	−8.3 a −2.3
		2003–2012	−7.5*	−9.3 a −5.8			
	Tocantins	2001–2008	0.6	−1.9 a 3.1	2001–2012	−3.3*	−5.5 a −1.1
		2008–2012	−9.7*	−15.2 a −4.0			
	Maranhão	2001–2004	4.5	−5.0 a 15.0	2001–2012	−3.4*	−5.8 a −1.0
		2004–2012	−6.3*	−8.3 a −4.2			
	Mato Grosso	2001–2012	−5.2*	−6.2 a −4.3	2001–2012	−5.2*	−6.2 a −4.3
	Other states of Brazil	2001–2003	6.3	−1.4 a 14.6	2001–2012	−4.0*	−5.2 a −2.8
		2003–2012	−6.2*	−6.9 a −5.5			
Incidence rates of leprosy in children <15 years per 100,000 inhabitants	Aggregated of municipalities	2001–2003	6.0	−10.1 a 24.9	2001–2012	−3.6*	−6.2 a −1.0
		2003–2012	−5.6*	−7.2 a −4.1			
	Rondônia	2001–2012	−3.0	−7.5 a 1.7	2001–2012	−3.0	−7.5 a 1.7
	Pará	2001–2012	−5.3*	−6.9 a −3.7	2001–2012	−5.3*	−6.9 a −3.7
	Tocantins	2001–2012	−0.9	−4.0 a 2.3	2001–2012	−0.9	−4.0 a 2.3
	Maranhão	2001–2012	−3.5*	−5.6 a −1.4	2001–2012	−3.5*	−5.6 a −1.4
	Mato Grosso	2001–2012	−5.9*	−7.8 a −3.9	2001–2012	−5.9*	−7.8 a −3.9
	Other states of Brazil	2001–2003	7.9	−1.7 a 18.5	2001–2012	−2.7*	−4.2 a −1.2
		2003–2012	−4.9*	−5.8 a 4.0			
Rates of new cases with grade-2 disabilities per 100,000 inhabitants	Aggregated of municipalities	2001–2012	−1.3	−2.6 a 0.1	2001–2012	−1.3	−2.6 a 0.1
	Rondônia	2001–2003	52.1	−33.8 a 249.0	2001–2012	0.9	−11.3 a 14.8
		2003–2012	−7.8*	−13.9 a −1.4			
	Pará	2001–2012	−2.2	−4.4 a 0.1	2001–2012	−2.2	−4.4 a 0.1
	Tocantins	2001–2012	−0.4	−3.0 a 2.2	2001–2012	−0.4	−3.0 a 2.2
	Maranhão	2001–2004	−0.4	−2.8 a 2.1	2001–2012	−0.4	−2.8 a 2.1
	Mato Grosso	2001–2012	−0.3	−2.7 a 1.9	2001–2012	−0.3	−2.7 a 1.9
	Other states of Brazil	2001–2008	0.2	−2.0 a 2.5	2001–2012	−2.6*	−4.3 a −0.9
		2008–2012	−7.4*	−11.3 a −3.2			

*Significantly different from 0 (*p* <0.05)

95 % CI: 95 % confidence intervals

In the aggregated of municipalities, the incidence rate in children under 15 years decreased from 24.32 to 15.79 new cases per 100,000 children between 2001 and 2012 and this reduction was statistically significant from 2003 to 2012 (APC: −5.6 %, 95 % CI: −7.2 to −4.1 %). There was also statistically significant reduction in the state of Pará between 2001 and 2012 (APC: −5.3 %, 95 % CI: −6.9 to −3.7 %) and in the states of Maranhão and Mato Grosso between 2001 and 2012 (APC: −3.5 %, 95 % CI: −5.6 to −1.4 % and APC: −5.9 %, 95 % CI: −7.8 to −3.9 %, respectively). On the other hand, the incidence rate of leprosy among children under 15 years remained stable in the states of Rondônia (APC: −3.0 %, 95 % CI: −7.5 to 1.7 %) and Tocantins (APC: −0.9 %, 95 % CI: −4.0 to 2.3 %) between 2001 and 2012 (Tables 1 and 2).

The rate of new cases with grade 2 disability was stable during the period, varying from 3.62 cases per 100,000 inhabitants in 2001 to 3.41 cases per 100,000 inhabitants in 2012. Statistically significant reduction only occurred in the state of Rondônia (APC: −7.8 %, 95 % CI: −13.9 to −1.4 %) between 2003 and 2012, and in municipalities located in other Brazilian states (not included in the aggregated data) (APC: −7.4 %, 95 % CI: −11.3 to −3.2 %) between 2008 and 2012 (Tables 1 and 2). Value inconsistency for this indicator in the year 2007 can be seen in Table 1, justifying its exclusion from the trend analysis as described in the methods section.

**Fig. 2:**
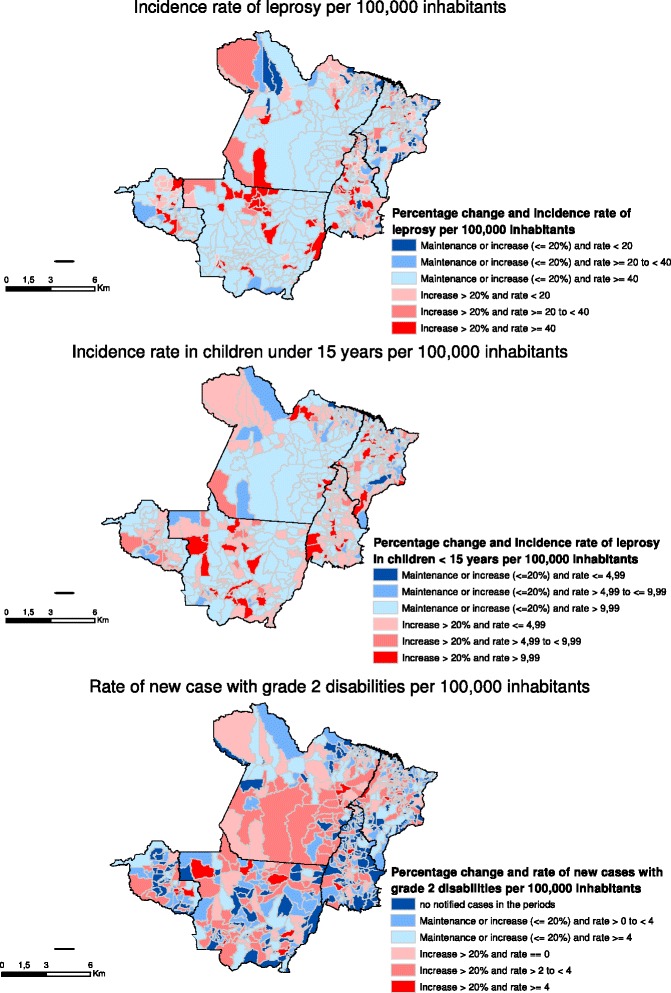
Maps of the percentage change in the epidemiological indicators of leprosy, periods 2001–2003 and 2010–2012

Figure 2 shows the change in the different study indicators, stratified by the initial rates of the baseline period (2001–2003). Thus, municipalities shown in red/pink scale are those that showed an increased (>20 %) of the rate, whereby red is the worst situation (because these municipalities presented high rates in the periods 2001–2003 and 2010–2012). Municipalities shown in blue are those where the rate reduced, remained the same or only increased slightly (no more than 20 %), whereby dark blue is the best situation (for those municipalities had low rates in 2001–2003 and yet still showed a reduction in 2010–2012).

Between 2001 and 2003, 404 (58.4 %) municipalities of the aggregated were considered to be hyperendemic with regard to the incidence rate of leprosy. There was an increase of more than 20 % in the rate in 63 (9.1 %) of these municipalities (highlighted in red - Fig. 2-a). During the same period, 288 (41.6 %) municipalities had moderate rates (low, medium or high) with regard to this indicator, and even so in 100 (14.5 %) municipalities percentage change was not greater than 20 % with regard to rate reduction, maintenance or increase (municipalities highlighted in dark blue – Fig. 2-a), when comparing the periods 2001–2003 and 2010–2012.

When analyzing the incidence rate in children under 15 years, 362 (52.3 %) municipalities were considered to be hyperendemic in the period 2001–2003. In 58 (8.4 %) of these municipalities the rate increased more than 20 % when comparing the periods 2001–2001 and 2010–2012 (municipalities highlighted in red – Fig. 2-b). With regard to this indicator, few municipalities (*n* = 9) had the best situation – low baseline rates and significant reduction in the period analyzed (highlighted in dark blue – Fig. 2-b).

With regard to rate of new cases with grade 2 disability, in the period 2001–2003, 51.6 % (357/692) of the municipalities did not notify grade 2 disability. 32.1 % (222/692) also had no notified cases in the period 2010–2012 (municipalities highlighted in dark blue – Fig. 2-c). On the other hand, in the period 2001–2003, the rate of new cases with grade 2 disability in 184(26.6 %) was considered to be very high (≥4 per 100,000 inhabitants). Of these, the increase in this rate in 19 (2.8 %) municipalities was >20 % when comparing 2001–2003 and 2010–2012 (municipalities highlighted in red - Fig. 2-c).

## Discussion

In the period 2001–2012, the aggregated municipalities analysed in this study accounted for 34.6 % of new leprosy cases in just 10 % of the Brazilian population. In general, trend analysis of the aggregated data showed statistically significant reduction in the incidence rate of leprosy and in the incidence rate in children under 15 years, although rates behaved differently between the states. On the other hand, the rate of new cases with grade 2 disability was stable during the period – suggesting late diagnosis – with continuing occurrence of cases with deformities or disabilities resulting from the disease in this studied aggregate.

Despite the reduction in the incidence rate of leprosy observed in this study, there are local hotspots of the disease in the country and thus maintaining disease in areas with high risk of transmission. Indeed, with regard to the leprosy incidence rate in Brazil between 2010 and 2012, 58.5 % (404/692) of the municipalities in the studied area remained hyperendemic. A possible explanation for the identification of local hotspots of the disease may be the existence of non-human or environmental sources of *Mycobacterium leprae* [16–18].

Our study also found a relevant reduction in the incidence rate in children under 15 years. It is noteworthy that this rate has been used as an important indicator of active disease transmission. A study conducted in Zambia (Africa) using data covering the period 1991–2010 found there is active leprosy transmission among children. The authors highlight that in addition to serious shortfalls in the country’s leprosy control programme, active transmission of the disease in children and late case diagnosis are still a cause for public health concern [19]. The incidence rate in children under 15 years in Brazil in 2012 was 4.8 new cases per 100,000 children. The aggregated data of our study found 22.2 new cases per 100,000 children. Although there are trends towards reduction, this result outlines a scenario of continuing transmission of the disease in the region studied and confirms that these aggregated municipalities form an area of high leprosy endemicity [20, 21].

Despite the falls in leprosy incidence in this aggregated study, the rate of new cases with grade 2 disability remained stable throughout the 12 year study period. This indicator has been used instead of leprosy prevalence owing to its being a strong marker for mapping more severe cases of the disease and it’s not being influenced by operational factors [7]. The stability in the rate of new cases with grade 2 disability noted in this study was similar to that found in other studies [22–24]. This result may indicate some shortcomings in the control of the disease, such as failure to diagnose early and failure to accompany and/or monitor cases. The apparent inconsistency in the stability of this indicator given the reduction in the trends of incidence rate of leprosy and among children under 15 years may be related to detection bias and this will be discussed below.

This study has some limitations inherent to studies conducted using secondary data. In particular with regard to leprosy, case underreporting is expected given that the disease begins in an insidious manner and its symptoms have multiple manifestations following a long asymptomatic/oligosymptomatic stage, and at times requires specific health professional training and experience for diagnosis [25]. Incidence rates of leprosy therefore only take into account “detected” cases of the disease and underreporting is more likely during the disease’s initial stage. Underreporting of cases is expected especially in areas of poor access to health services. These factors may bias differently the study indicators, and the trends in states analyzed. This fact may justify, at least partly, the stability observed in rate of new cases with grade 2 disability (when underreporting may be rarer) and the reduction in incidence rates of leprosy (where a high level of under-reporting is expected). Moreover, leprosy has a complex transmission chain and this hinders the establishment of clear epidemiological links to support the diagnosis stage [26, 27].

Another limitation relates to the change in rate of new case with grade 2 disability case definition criteria which had an impact on notifications made on the information system in 2007. With the aim of minimizing this limitation in this study, 2007 data was excluded given that it could influence the analysis of this indicator’s trends.

Adequate multidrug therapy (MDT) and early and accurate case diagnosis continue to be the main strategies for leprosy control [1, 28]. Nevertheless, based on the combination of biological and epidemiological evidence, Yadav and collaborators suggest that leprosy cannot be eliminated only through MDT, given that the disease’s microbiology is not yet totally understood [2]. New forms of control and detection are needed to combat transmission more directly. One approach which may be promising involves chemoprophylaxis in campaigns in schools located in high risk areas, as well as being targeted at groups such as those who have contact with the disease in their households [6, 29]. Indeed, recently the Brazilian Ministry of Health has been implanting strategies of this nature, the evaluation of which may provide insight into their effectiveness [3].

## Conclusion

Despite the reduction in the leprosy incidence rate, the rate of new cases with grade 2 disability was stable during the study period. These findings suggest delays in case diagnosis and shortcomings in preventing disabilities, highlighting possible operational difficulties in controlling the disease. Therefore, there is a need to improve control strategies with the aim of preventing grade 2 disability cases, especially in hyperendemic municipalities, in order to reduce disease burden and prevent disabilities.

## Abbreviations

AAPC: Average annual percentage change
APC: Annual percentage change
BCG: Bacillus Calmette-Guérin
CAAE: Presentation of Certificate for Ethics Assessment
CI: Confidence interval
Datasus: Informatics Department of the Brazilian National Health System
ESRI: Environmental Systems Research Institute
IBGE: Brazilian Institute of Geography and Statistics
MDT: Multidrug therapy
SINAN: Notifiable diseases information system
